# The induced by an electromagnetic field coexistence of types I and II spectra in Weyl semimetals

**DOI:** 10.1038/s41598-018-32104-y

**Published:** 2018-09-12

**Authors:** Zaur Z. Alisultanov

**Affiliations:** 1 Amirkhanov Institute of Physics, Russian Academy of Sciences, Dagestan Science Centre, Makhachkala, Russia; 2 Dagestan State University, Makhachkala, Russia

## Abstract

Due to their unique properties, Weyl semimetals (WSMs) are promising materials for the future electronics. Currently, the two types (I and II) of WSMs are discovered experimentally. These types of WSMs differ from each other in their topological properties. In this paper we showed that a coexistence of types I and II Weyls spectra is possible in some WSMs under crossed magnetic and electric fields. This is possible in systems with non-equivalent Weyl points (WPs). In particular, it is possible in strained WSMs. Such phase, controlled by electromagnetic field, is principally new for topological matter physics. It is obvious, that in this regime new features of electron transport will appear. We showed that this effect can also be considered as a mechanism of strain induced type-I-type-II transition.

## Introduction

Weyl semimetals (WSMs) are one of the main topics in today’s physics of condensed matter^1–11^. These topological materials are promising for new electronics due to their unique properties. Study of the influence of external perturbations on the properties of WSMs is interesting not only from an applied point of view. Such studies provides helpful information about the fundamental problems of high-energy physics. As examples of such perturbations can be called an electromagnetic field, elastic deformations of crystal lattice, etc. Particularly, Dirac and Weyl systems have the interesting relativistic features in crossed magnetic and electric fields^12–20^. Landau levels (LLs) (and accordingly, all thermodynamic phenomena that related with them) in such systems can be essentially changed by an electric field. This is related to the fact that a cyclotron mass of carriers depends on the energy in the case of Dirac-like spectrum. Recently, Landau quantization problem in WSMs under crossed fields have been investigated^21–27^. A particularly interesting phenomenon that occurs in crossed fields is the collapse of LLs, when these levels disappear. Crossed fields lead to the drift of charged carriers. Relativistic effects occur when the velocity of this drift approaches the Fermi velocity. The other interesting phenomenon (see below) is *the induced by an electromagnetic field transition between different types of spectrum in WSMs*. There are at least two reasons for such researches. On the one hand, the electric field (of external or other origin) can be used as an effective tool for changing the diamagnetic properties of Dirac-like systems. On the other hand, the fact that LLs depend on the electric field should be included to a rigorous theories of phenomena which occur in the regime of crossed fields (the galvanomagnetic effects, for example). In this paper we show some new manifestations of crossed-fields regime.

**Figure 1:**
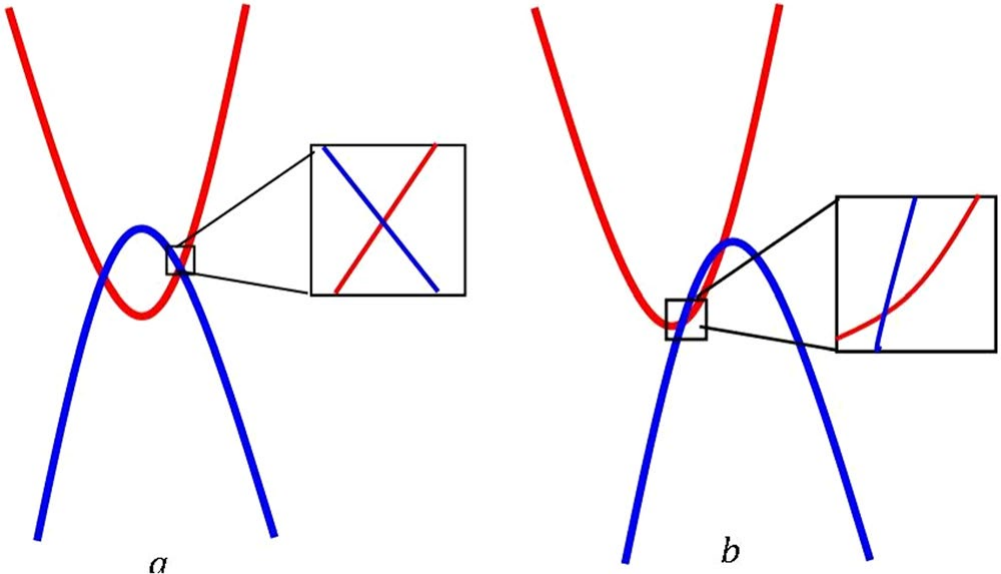
Type-I (**a**) and type-II (**b**) Weyl spectra.

Figure 1(a) shows one of the possibilities of simple formation of the Weyl spectrum. Such spectrum is direct and Weyl Hamiltonian at the intersection points (called WPs) has the form

1 $$\hat{ {\mathcal H} }=\pm {\upsilon }_{F}{\boldsymbol{\sigma }}{\bf{p}}\mathrm{.}$$

The signs “±” denote the chirality of carriers at different WPs, which are localized at **k**_+_ and **k**_−_. Respectively, **p** = $\hslash$(**k** − **k**_+_) for *W*_+_ and **p** = $\hslash$(**k** − **k**_−_) for *W*_−_. *υ*_*F*_ is the Fermi velocity of carriers, ***σ*** = *σ*_*x*_, *σ*_*y*_, *σ*_*z*_ are the Pauli matrices. The spectrum of the Hamiltonian (1) has the form: *ε* = ±*υ*_*F*_|*p*|. Such WPs are stable, due to the fact that the Hamiltonian (1) contains all three Pauli matrices.

Besides of the WSMs with the direct spectrum of type-I, the WSMs with an tilted spectrum corresponding to type-II is possible. Such a spectrum is formed in crystal at the intersection of the Fermi pockets (Fig. 1(b)). This situation is described by the following Hamiltonian

2 $$\hat{ {\mathcal H} }=\pm {\upsilon }_{F}{\boldsymbol{\sigma }}{\bf{p}}+{\boldsymbol{\omega }}{\bf{p}},$$

where ***ω*** = (*ω*_*x*_, *ω*_*y*_, *ω*_*z*_) is the tilt parameter. The condition ${\upsilon }_{F}^{2} > {\omega }^{2}$ corresponds to the type-I WSMs with a tilted spectrum, and at ${\upsilon }_{F}^{2} < {\omega }^{2}$ the Hamiltonian describes the type-II WSMs. In the general case, the parameter ***ω*** has different values for different WPs. To denote this fact, we will modify the tilt parameter by adding the index: ***ω***^*η*^ and *η* = ±1 for the *W*_±_ (below, we will consider only one of the WPs pairs, suggesting that our results can be easily generalized to all other pairs). The type-II WSMs has been proposed recently in^6–9^. The compounds WTe_2_ and MoTe_2_ are the materials representing the family of type-II WSMs. It was shown that WTe_2_ is the fermion system of a new type with a strong violation of Lorentz invariance. It was shown that WTe_2_ contains eight WPs in the Brillouin zone, while MoTe_2_ is characterized by four WPs. Topological phase transitions between different types of WSMs were discussed in detail in a recent paper^28^.

The WSMs of different types have different topological and transport properties. Besides pure type-I and type-II WSMs, there exists a novel type, dubbed “hybrid Weyl semimetal”^29^, which contains the both types of Weyl points. Such WSMs can be called - type-3/2 WSMs. Recent studies of transport properties of these hybrid WSMs^30,31^ show that in such materials the anomalous Hall transport is realized. In this paper we demonstrate theoretically that crossed magnetic and electric fields can induce a coexistence of type-I and type-II Landau spectra. I.e. the pure type-I (or pure type-II) WSM can be switched to regime of hybrid WSM using crossed fields. The point is, that in crossed magnetic and electric fields the drift of electrons appears. This leads to changing of tilt parameter in the drift direction. As a consequence, the total tilt parameter will change: ${\boldsymbol{\omega }}\to \tilde{{\boldsymbol{\omega }}}$. In type-I WSMs, ${\upsilon }_{F}^{2} > {\omega }^{2}$ in the absence of those fields. In the presence of fields-induced drift, this relation can be changed to ${\upsilon }_{F}^{2} < {\tilde{\omega }}^{2}$ that corresponds to type-II WSMs. Note, that this effect is absent in magnetic field. The tilt parameter changes due to the drift velocity which appears under crossed fields. I.e. an electric field must be also present. Thus, *the transition between type-I and type-II can be induced by the electromagnetic field*. If WPs of pair are absolutely similar (***ω***^+^ = ***ω***^−^) then this transition will occur identically near both points. However, the picture of transition will change if the tilt parameters for different WPs differ from each other. Indeed, in this case a condition ${\upsilon }_{F}^{2}={\tilde{\omega }}^{2}$ will differ for different WPs. Then the situation can be realized when at some value of electric field a I-II transition will occur only at the one of WPs. At the same time, a spectrum near the second WP still corresponds to type-I. Thus, *the coexistence of types I and II Weyl spectra can be induced*. Such phase, controlled by electromagnetic field, is principally new for topological matter physics. It is obvious that in this regime, the new features of electron transport will appear.

## Results and Discussions

The Hamiltonian (2) gives the following expression for the energy spectrum:

3 $$\varepsilon =\pm \,{\upsilon }_{F}|p|+\mathop{{\boldsymbol{\omega }}}\limits^{\eta }{\bf{p}}$$

**Figure 2:**
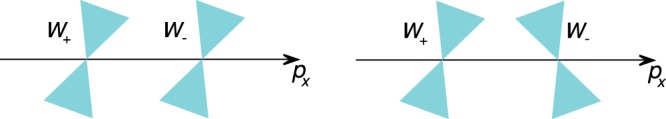
Weyl cones for two cases: ${\mathop{\omega }\limits^{+}}_{x}={\mathop{\omega }\limits^{-}}_{x}$ (left) and ${\mathop{\omega }\limits^{+}}_{x}=-\,{\mathop{\omega }\limits^{-}}_{x}$ (right).

Such WPs are still topologically protected. Indeed, the perturbation in the form of a unit matrix leads only to a displacement of the WPs with respect to energy and momentum axes: $\hat{ {\mathcal H} }\to \hat{ {\mathcal H} }+I{U}_{0}\Rightarrow E\to E(p)+{U}_{0}$. As we can see, the gap does not open. On the other hand, the perturbation in the form of Pauli matrices $\hat{ {\mathcal H} }\to \hat{ {\mathcal H} }+{\boldsymbol{\sigma }}{\bf{U}}$ leads to the following spectrum $\varepsilon =\mathop{{\boldsymbol{\omega }}}\limits^{\eta }{\bf{p}}\pm {\upsilon }_{F}\sqrt{|{\bf{p}}+{\bf{U}}/{\upsilon }_{F}{|}^{2}}$. This spectrum is also shifted relatively to the original spectrum (3), but does not contain a bandgap. As we noted in the introduction, in the general case, the tilt parameter has different values for different WPs. As an example, the two different cases of WSM are shown in Fig. 2. The condition ${\upsilon }_{F}^{2}={\mathop{\omega }\limits^{\eta }}^{2}$ corresponds to the phase transition point between type-I and type-II WSMs. This transition can be attributed to the family of Lifshitz-like phase transitions^28^. The DoS has a singularity at this phase transition point. Indeed, the DoS for the spectrum (3) has the following form (see^22,23^)

4 $${\rho }_{0}=\frac{1}{{(2\pi \hslash )}^{2}}\frac{{\upsilon }_{F}}{{({\upsilon }_{F}^{2}-{\mathop{\omega }\limits^{\eta }}^{2})}^{2}}\frac{{\varepsilon }^{2}}{\hslash }$$

As we can see, point ${\upsilon }_{F}^{2}={\mathop{\omega }\limits^{\eta }}^{2}$ is the pole of DoS. This is related with the following. At ${\upsilon }_{F}^{2}={\mathop{\omega }\limits^{\eta }}^{2}$, one of the spectrum branches (one edge of Weyl cone) becomes horizontal. In the continuous model this means that the DoS becomes infinitely large. However, in the framework of lattice model, the momentum values are limited by *π*$\hslash$/*a* (*a* is the lattice constant). Thus, in the framework of lattice model the DoS at ${\upsilon }_{F}^{2}={\mathop{\omega }\limits^{\eta }}^{2}$ is large, but not infinite. In presence of magnetic field we should replace **p** → ***π*** = **p** + *e*/*c***A**. Then

5 $$\hat{ {\mathcal H} }=\pm \,{\upsilon }_{F}{\boldsymbol{\sigma }}{\boldsymbol{\pi }}+\mathop{{\boldsymbol{\omega }}}\limits^{\eta }{\boldsymbol{\pi }}$$

Using the Landau gauge and the double Lorentz boost $\hat{ {\mathcal H} }\to {e}^{\frac{{\sigma }_{x}{\lambda }_{1}}{2}\frac{{\sigma }_{y}{\lambda }_{2}}{2}}\hat{ {\mathcal H} }{e}^{\frac{{\sigma }_{x}{\lambda }_{1}}{2}\frac{{\sigma }_{y}{\lambda }_{2}}{2}}$, where $\tanh \,{\lambda }_{1}={\mathop{\omega }\limits^{\eta }}_{x}/{\upsilon }_{F}$ and $\tanh \,{\lambda }_{2}={\mathop{\omega }\limits^{\eta }}_{y}/{\upsilon }_{F}$, we obtain the following expression for LLs (see^25,26^)

6 $${\varepsilon }_{n}^{\eta }={\rm{sgn}}(n){\upsilon }_{F}\sqrt{2{\gamma }_{0}^{3}{l}_{H}^{-2}{\hslash }^{2}n+{\gamma }_{0}^{2}{p}_{z}^{2}}+{\mathop{\omega }\limits^{\eta }}_{z}{p}_{z},\,n\ne \mathrm{0,}$$

7 $${\varepsilon }_{0}^{\eta }=(\eta {\upsilon }_{F}{\gamma }_{0}+{\mathop{\omega }\limits^{\eta }}_{z}){p}_{z},\,n=\mathrm{0,}$$

**Figure 3:**
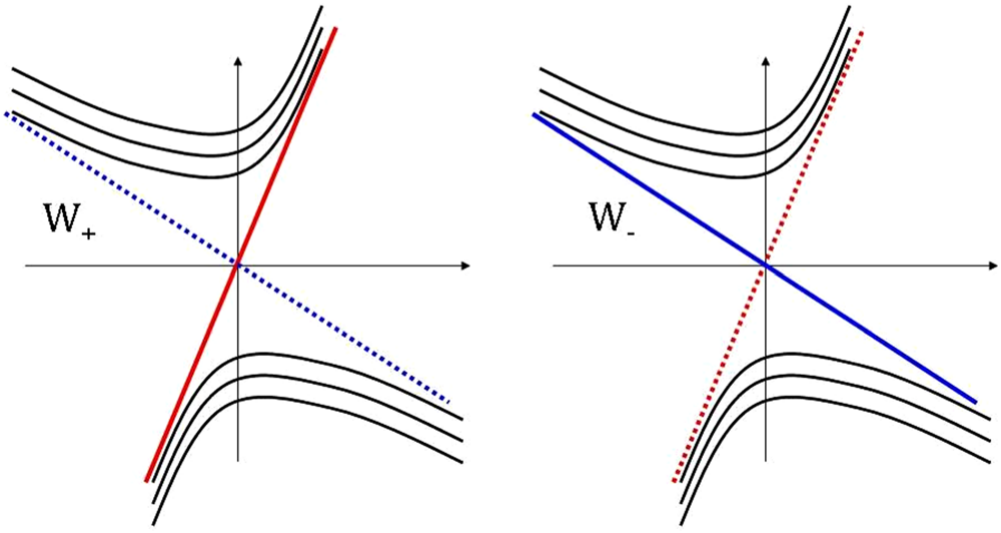
Type-I Landau levels *ε*_*n*_ as function of *p*_*z*_ at *W*_+_ (left) and *W*_−_ (right).

**Figure 4:**
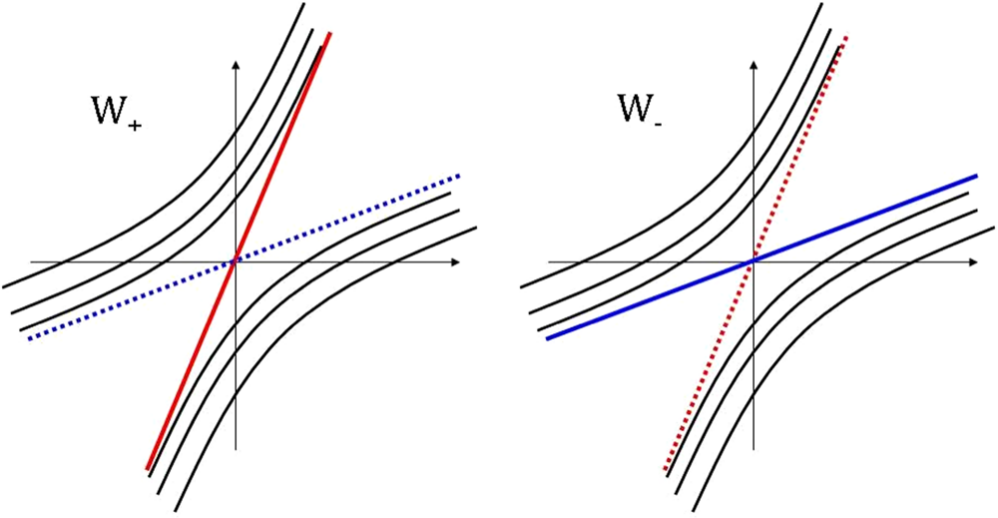
Landau levels *ε*_*n*_ as function of *p*_*z*_ in type-II WSMs near WPs: *W*_+_ (left) and *W*_−_ (right).

**Figure 5:**
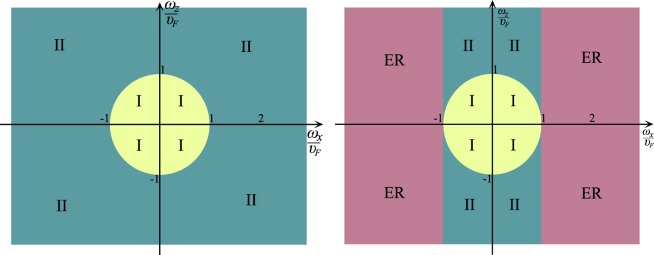
WSM diagram in $(\frac{{\omega }_{x}}{{\upsilon }_{F}},\frac{{\omega }_{z}}{{\upsilon }_{F}})$ plane without magnetic field (left) and under magnetic field (right). The pink region corresponds to electric regime (ER).

In the last equations ${\gamma }_{0}=\sqrt{1-\frac{{\mathop{\omega }\limits^{\eta }}_{x}^{2}+{\mathop{\omega }\limits^{\eta }}_{y}^{2}}{{\upsilon }_{F}^{2}}}$. Note, that the magnetic field doesn’t affect the type of spectrum. As in the absence of magnetic field, the difference between two types is defined only by the ratio: ${\upsilon }_{F}^{2}$ and *ω*^*η*2^. A qualitative picture of LLs in type-I WSM (${\upsilon }_{F}^{2}{\gamma }_{0}^{2} > {\mathop{\omega }\limits^{\eta }}_{z}^{2}$) is shown in Fig. 3. For the case of type-II WSMs (${\upsilon }_{F}^{2}{\gamma }_{0}^{2} > {\mathop{\omega }\limits^{\eta }}_{z}^{2}$) Landau levels are shown in Fig. 4. WSM diagram in $(\frac{{\omega }_{x}}{{\upsilon }_{F}},\frac{{\omega }_{z}}{{\upsilon }_{F}})$ plane is given in Fig. 5.

Let’s consider the Landau quantization problem in WSMs at the presence of the electric field. Recently, the similar problem has been considered in few papers^21–23,25–27^. We consider WSM under magnetic **H** = (0, 0, *H*) and electric **E** = (0, *E*, 0) fields. The Landau quantization problem solution for the case of WSM with tilted spectrum under such field configuration has the following form (we used the double Lorentz boost $\hat{ {\mathcal H} }\to {e}^{\frac{{\sigma }_{x}{\lambda }_{1}}{2}\frac{{\sigma }_{y}{\lambda }_{2}}{2}}\hat{ {\mathcal H} }{e}^{\frac{{\sigma }_{x}{\lambda }_{1}}{2}\frac{{\sigma }_{y}{\lambda }_{2}}{2}}$, where $\tanh \,{\lambda }_{1}=({\upsilon }_{0}-{\mathop{\omega }\limits^{\eta }}_{x})/{\upsilon }_{F}$ and $\tanh \,{\lambda }_{2}={\mathop{\omega }\limits^{\eta }}_{y}/{\upsilon }_{F}$)

8 $${\varepsilon }_{n}^{\eta }={\rm{sgn}}(n){\upsilon }_{F}\sqrt{2{\gamma }_{\eta }^{3}{l}_{H}^{-2}{\hslash }^{2}n+{\gamma }_{\eta }^{2}{p}_{z}^{2}}+{\mathop{\omega }\limits^{\eta }}_{z}{p}_{z}+{\upsilon }_{0}{p}_{x},\,n\ne \mathrm{0,}$$

9 $${\varepsilon }_{0}^{\eta }=(\eta {\upsilon }_{F}{\gamma }_{\eta }+{\mathop{\omega }\limits^{\eta }}_{z}){p}_{z}+{\upsilon }_{0}{p}_{x},\,n=\mathrm{0,}$$

where ${\gamma }_{\eta }=\sqrt{1-\frac{{({\upsilon }_{0}-{\mathop{\omega }\limits^{\eta }}_{x})}^{2}+{\mathop{\omega }\limits^{\eta }}_{y}^{2}}{{\upsilon }_{F}^{2}}},{{\boldsymbol{\upsilon }}}_{0}=c[{\bf{E}}{\bf{H}}]/{H}^{2}$. One can see that at ${\upsilon }_{F}^{2}{\gamma }_{\eta }^{2} < {\mathop{\omega }\limits^{\eta }}_{z}^{2}$ the LLs (8) corresponds to type-I WSMs (see Fig. 3). From the other hand, when ${\upsilon }_{F}^{2}{\gamma }_{\eta }^{2} > {\mathop{\omega }\limits^{\eta }}_{z}^{2}$ we have LLs of type-II WSMs (see Fig. 4). Thus, the condition ${\upsilon }_{F}^{2}{\gamma }_{\eta }^{2}={\mathop{\omega }\limits^{\eta }}_{z}^{2}$ corresponds to the phase transition point between type-I and type-II WSMs. This phase transition occurs at ${\upsilon }_{0}=-\,\sqrt{{\upsilon }_{F}^{2}-{\mathop{\omega }\limits^{\eta }}_{y}^{2}-{\mathop{\omega }\limits^{\eta }}_{z}^{2}}+{\mathop{\omega }\limits^{\eta }}_{x}$.

DoS at the presence of crossed fields has the following expression (see^27^)

10 $$\rho (\varepsilon )={\rho }_{0}(\varepsilon )+{\rho }_{osc}(\varepsilon )$$

where

11 $${\rho }_{0}=\frac{1}{{\mathrm{(2}\pi \hslash )}^{2}}\frac{{\upsilon }_{F}}{{({\upsilon }_{F}^{2}{\gamma }_{\eta }^{2}-{\mathop{\omega }\limits^{\eta }}_{z}^{2})}^{2}}\frac{{\varepsilon }^{3}-{(\varepsilon -eU)}^{3}}{3eU\hslash }$$

12 $${\rho }_{osc}=\frac{1}{{\mathrm{(2}\pi )}^{2}}\frac{{\upsilon }_{F}{\gamma }_{\eta }^{\mathrm{3/2}}}{4\pi eU{l}_{H}^{3}\sqrt{{\upsilon }_{F}^{2}{\gamma }_{\eta }^{2}-{\mathop{\omega }\limits^{\eta }}_{z}^{2}}}\sum _{k\mathrm{=1}}\frac{1}{{k}^{2}}(\cos \,\pi (k{\xi }_{(\varepsilon -eU)}+\frac{1}{4})-\,\cos \,\pi (k{\xi }_{\varepsilon }+\frac{1}{4}))$$

where ${\xi }_{\varepsilon }^{2}=\frac{{l}_{B}^{2}}{{\hslash }^{2}}\frac{{\varepsilon }^{2}}{\gamma ({\upsilon }_{F}^{2}{\gamma }_{\eta }^{2}-{\mathop{\omega }\limits^{\eta }}_{z}^{2})},U=E{L}_{y}$. From the last expressions we can see that the DoS has singularity at ${\upsilon }_{F}^{2}{\gamma }_{\eta }^{2}={\mathop{\omega }\limits^{\eta }}_{z}^{2}$.

Thus, *an electric field can lead to a phase transition between I and II WSM types*.

Another important consequence of the previous results is related to the nonequivalence of WPs. In the case of nonequivalent WPs the quantity *γ*_*η*_ has different values for different WPs. This means that different values of |*υ*_0_| will correspond to the phase transition conditions for these points. Then, the situation when a phase transition occurs near only one of the WPs of the pair can be realized. In this case, the spectrum near one of the WPs corresponds to type II, while near the second point it corresponds to type I. Thus, a phase in which two types of Weyl fermions (WFs) coexist appears. For example, for the case shown in Fig. 2 we obtain

13 $${\gamma }_{+}=\sqrt{1-\frac{{({\upsilon }_{0}-{\omega }_{x})}^{2}+{\omega }_{y}^{2}}{{\upsilon }_{F}^{2}}},\,{\gamma }_{-}=\sqrt{1-\frac{{({\upsilon }_{0}+{\omega }_{x})}^{2}+{\omega }_{y}^{2}}{{\upsilon }_{F}^{2}}}\mathrm{.}$$

The difference between *γ*_+_ and *γ*_−_ leads to the difference between phase transition conditions for different WPs. For the case that we consider here, the phase transition in point *W*_+_ occurs at ${\upsilon }_{0}^{+}=-\,\sqrt{{\upsilon }_{F}^{2}-{\omega }_{y}^{2}-{\omega }_{z}^{2}}+{\omega }_{x}$. From the other hand, for point *W*_−_ we have ${\upsilon }_{0}^{-}=\sqrt{{\upsilon }_{F}^{2}-{\omega }_{y}^{2}-{\omega }_{z}^{2}}-{\omega }_{x}$. I.e. the transition I-II at given fields cannot occur in both WPs simultaneously. It is interesting that ${\upsilon }_{0}^{+}=-{\upsilon }_{0}^{-}$. This means that by changing the direction of magnetic or electric field (this leads to the change of sign of ***υ***_0_) we can change the types of Weyl fermions near the first or the second WPs. Thus, two different forms of hybrid WSMs can be realized. For example, in one of the forms, type-I corresponds to the first WP and type-II corresponds to the second one. For contrast, in another form, type-I corresponds to the second WP, while type-II corresponds to the first one. The induced by an electromagnetic field the phase transition and the coexistence of types I and II spectra are the main results of this work, which is absent in the previous literature.

**Figure 6:**
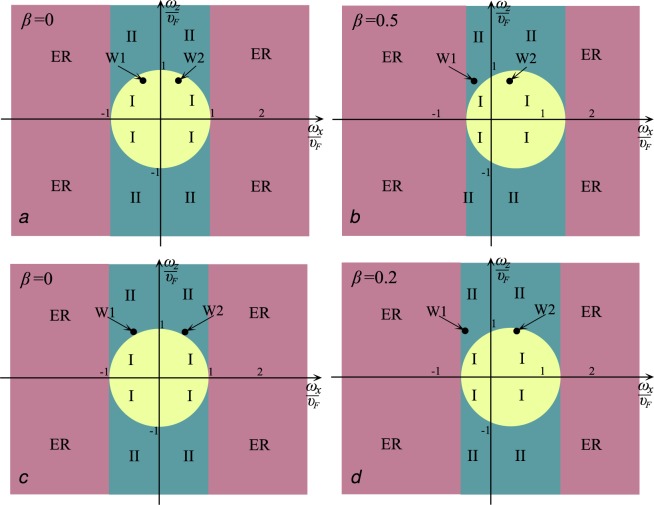
The simplest demonstration of transition in $(\frac{{\omega }_{x}}{{\upsilon }_{F}},\frac{{\omega }_{z}}{{\upsilon }_{F}})$ plane. Top: type-I - type-II transition leads to coexistence of two types: in absence of the electric field both WPs are type-I without (**a**), but in presence of the electric field the transition to type-II occurs near one of the points (**b**). Bottom: type-II - type-I transition leads to coexistence of two types. In absence of the electric field both WPs are type-II (**c**), but in presence of the electric field the transition to type-I occurs near one of the points (**d**).

The simplest demonstration of type-I - type-II transition in $(\frac{{\omega }_{x}}{{\upsilon }_{F}},\frac{{\omega }_{z}}{{\upsilon }_{F}})$ plane is given in Fig. 6 (Top).

The crossed pseudo-electric and pseudo-magnetic fields in WSMs can appear at crystal strain. Then, the transition considered above can be called a strain-induced phase transition. The gauge pseudo-fields induced by the crystal lattice deformation^32^ are one of the attractive relativistic effects in the physics of Dirac-like systems. This phenomenon is an additional bridge between condensed matter physics and quantum field theory. Recently, these pseudo-fields have been studied in WSMs^33^. The different topological effects induced by such fields have been investigated in^34–39^. The DoS oscillations and Shubnikov-de Haas effect caused by a pseudo-magnetic field were investigated in^36^. In ref.^40^, the Landau quantization problem in WSM under crossed pseudo-magnetic and pseudo-electric fields have been considered. Indeed, it was shown that a lattice deformation induces both the pseudo-magnetic and the electric fields. The ref.^41^ is devoted to the study of this problem in graphene. The general and important property of such fields is that the pseudo-magnetic fields appear with opposite signs at the WPs with different chirality, while a signs of the electric field are the same. However, it has been reported in paper^42^ that an electric field can also have a pseudo-nature. This means, that, in the general case, the strain-induced electric field consists of a deformation potential and a pseudo-electric field that has the opposite signs at different WPs. This general case is not essential for our basic idea. Therefore, we consider here the case of a pure deformation potential.

Elastic deformations are defined by a strain tensor: *u*_*ij*_ = 1/2(∂_*i*_*u*_*j*_ + ∂_*j*_*u*_*i*_), where *u*_*i*_ is a displacement vector. Vector and scalar potentials of strain-induced fields are determined by the strain tensor as follows:

14 $${A}_{i}={u}_{ij}{b}_{j},\Phi =g\sum _{i}{u}_{ii}$$

where **b** = (*b*_*x*_, *b*_*y*_, *b*_*z*_) is the distance between WPs in momentum space, *g* is the coupling constant associated with the deformation potential.

To demonstrate the main idea, we consider a bending of thin film of type-I WSM with absolutely identical tilt parameters near different WPs.

We use the model of a rectangular lattice, similarly to ref.^40^. We denote by *b* the distance between the WPs along the axis *k*_*x*_ in momentum space. The bend deformation, that we consider here, is described by the following displacement vector components

15 $$\begin{array}{rcl}{u}_{x} & = & {u}_{0}\mathrm{(2}xy+Cx),\\ {u}_{y} & = & {u}_{0}(\,-\,{x}^{2}-Dy(y+C),\\ {u}_{z} & = & \mathrm{0,}\end{array}$$

where *u*_0_, *C*, *D* are the constants; see details in^40^. Such strain induces a homogeneous pseudo-magnetic and deformation electric fields. Indeed, the formula (14) give the following expressions for a vector potential and scalar potentials: ***A*** = (*u*_0_(2*y* + *C*)*b*, 0, 0) and Φ(*y*) = *u*_0_(1 − *D*)(2*y* + *C*). Below we use more simply expressions for the potentials *A*_*x*_ = 2*u*_0_ *by* ≡ −*H*_0_*y* and Φ(*y*) = 2*u*_0_(1 − *D*)*y* ≡ −*E*_0_*y*. At the presence of the pseudo-magnetic field we have **p** → ***π***_*η*_ = **p** + *ηe*/*c***A**. Thus, resulting Hamiltonian of the strained WSM have the following form

16 $$H=\eta {\upsilon }_{F}{\boldsymbol{\sigma }}{{\boldsymbol{\pi }}}_{\eta }+{\boldsymbol{\omega }}{{\boldsymbol{\pi }}}_{\eta }+e{E}_{0}y\mathrm{.}$$

Such a configuration of fields completely corresponds to the problem considered above (see Eqs (8) and (9). Therefore, the eigenvalues of Hamiltonian (16) are given by Eq. (8) where ${\gamma }_{\eta }=\sqrt{1-\frac{{({\upsilon }_{0}-{\omega }_{x})}^{2}+{\omega }_{y}^{2}}{{\upsilon }_{F}^{2}}}$ and *υ*_0_ = *ηcE*_0_/*H*_0_. Note that *υ*_0_ = *cE*_0_/*H*_0_ for *W*_+_ and *υ*_0_ = −*cE*_0_/*H*_0_ for *W*_−_. Then ${\gamma }_{\eta }=\sqrt{1-\frac{{(|{\upsilon }_{0}|-\eta {\omega }_{x})}^{2}+{\omega }_{y}^{2}}{{\upsilon }_{F}^{2}}}$. Thus, due to the fact that the coefficients *γ*_+_ and *γ*_−_ differ for different WPs, the phase transition condition in strained WSMs is not satisfied for both WPs. I.e. the I-II transition occurs only near one of WPs.

Above we have considered a type-I-type-II phase transition, which can be induced by an electric field (external or strain-induced). At the same time, the opposite situation is also possible when electric field induces the transition from type-II to type-I. Let’s give a description of such a situation in a general form for a single WP. Let ${\upsilon }_{F}^{2} < {\omega }^{2}$ for some WP. Then LLs are described by expression ([eq: 6]) where ${\gamma }_{0}^{2}{\upsilon }_{F}^{2} < {\omega }_{z}^{2}$. In presence of the electric field we have ${\gamma }_{0}^{2}\to {\gamma }^{2}=1-\frac{{({\omega }_{x}-{\upsilon }_{0})}^{2}+{\omega }_{y}^{2}}{{\upsilon }_{F}^{2}}$. Then we have ${\gamma }^{2} > {\gamma }_{0}^{2}$ for *ω*_*x*_ > 0 and ${\gamma }^{2} < {\gamma }_{0}^{2}$ for *ω*_*x*_ > 0. This means that at some value of *υ*_0_ the condition ${\gamma }^{2}{\upsilon }_{F}^{2} > {\omega }_{z}^{2}$ can be achieved in one of the WPs. If *υ*_0_ > 0 then the transition from type-II to type-I can occur in the WP with *ω*_*x*_ > 0. At the same time, in the WP with *ω*_*x*_ > 0 the type-II the requirement ${\gamma }_{0}^{2}{\upsilon }_{F}^{2} < {\omega }_{z}^{2}$ will be still satisfactory. Thus, the electric field can induce the type-II-type-I transition in one of the WPs. The diagram for this case is given in Fig. 6 (Bottom).

## Conclusion

The present paper demonstrates that in the tilted type-I WSM under the crossed magnetic and electric fields a coexistence of types I and II WFs can be realized. It is obvious that such a phase will be characterized by unusual transport properties. Indeed, in^30,31^ the authors considered Hall conductivity of the tilted WSMs with various values of tilt parameters near different WPs. The authors of ^30,31^ showed that anomalous Hall effect can occur at coexistence of type-I and type-II WFs.

Applicability of our results to the hybrid WSM is of great interest. It is obvious that the crossed fields regime will lead to the possibility to control the type of spectrum in the hybrid WSM. I.e. the electromagnetic field applied to hybrid WSM can induce transition to pure type (I or II).

Further, it is of great interest to investigate the effect of crossed fields on Fermi-arc states surface. The latter is the hallmark of the EP. Our results, which are valid for bulk states, can be generalized to surface states using the elegant model proposed in^43^. We expect that crossed fields can lead to the appearance of a new surface Fermi-arc state of type- 3/2. This type of Fermi-arc begins in type-I WP and ends in type-II WP.

The transition described above occurs at some values of fields. This means that field dependence of any thermodynamic or transport quantity will consist of two regions: a region where the types of both WPs are the same and a region where these types are different. Therefore, field dependence will contain the point where transition between normal and abnormal regions will occur. In particular, we expect such a transition in the field dependence of Hall conductivity. Another example where such a transition could be found is positive longitudinal magnetoconductivity induced by a chiral anomaly.

Finally, we should note the following. One of the interesting phenomena in WSM under crossed magnetic and electric fields is a collapse of LLs. This phenomenon was investigated for various cases. However, all these investigations corresponded to one WP. In the case of two non-equivalent WPs the collapse condition differs in various points. It can be realized a situation when collapse and transition to electric regime occur in one of the Weyl points only, while the another point will characterized by magnetic regime. Thus, the Landau levels can vanish in one of the Weyl points under the electromagnetic field. Such phase can demonstrate new topological properties.
